# Supplementary material for: Salsolinol-Containing Senna silvestris Exerts Antiviral Activity Against Hepatitis B Virus
Source: Plants (Basel). 2025 Aug 1;14(15):2372. doi: 10.3390/plants14152372 (PMC12349215; doi:10.3390/plants14152372)
Supplement: Supplementary file 1 [file plants-14-02372-s001.zip › plants-3615639-supplementary.pdf]

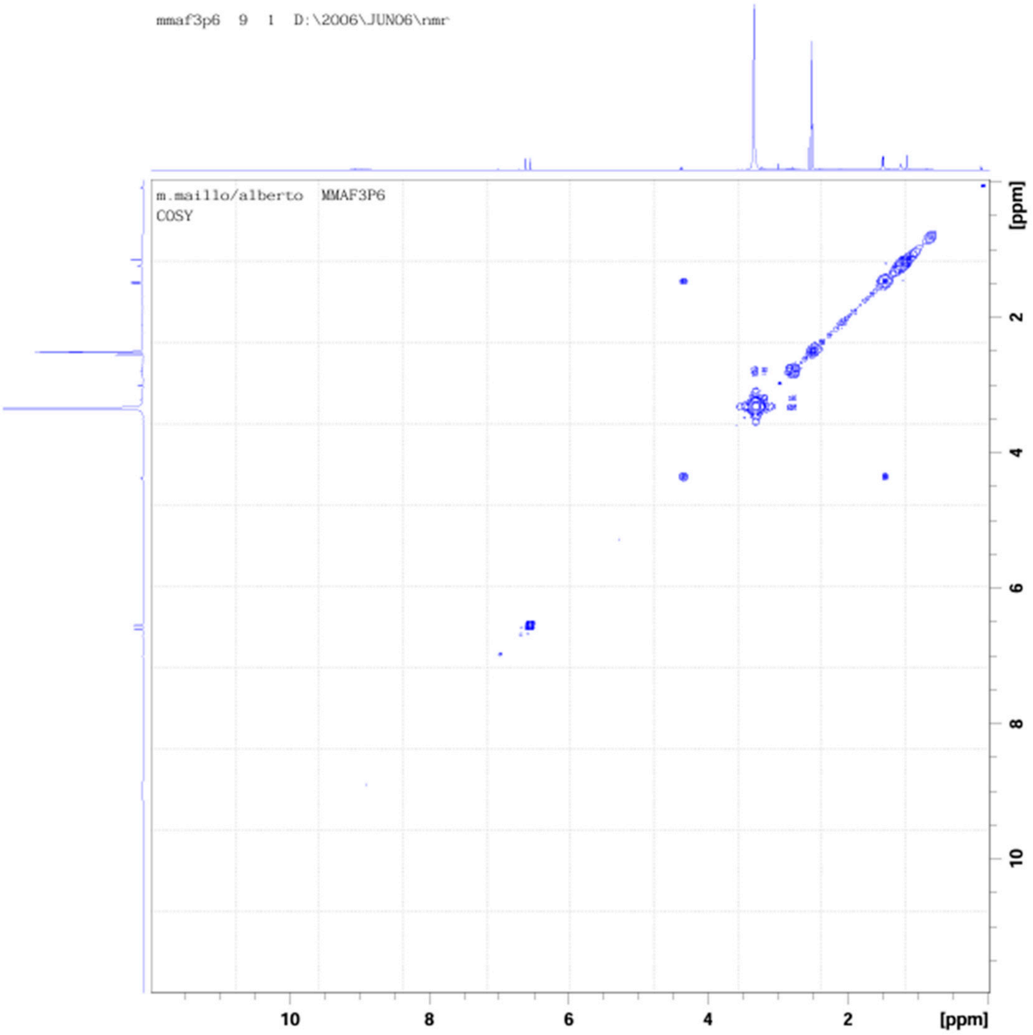

Figure S1. 2D COSY spectrum of G3 peak.

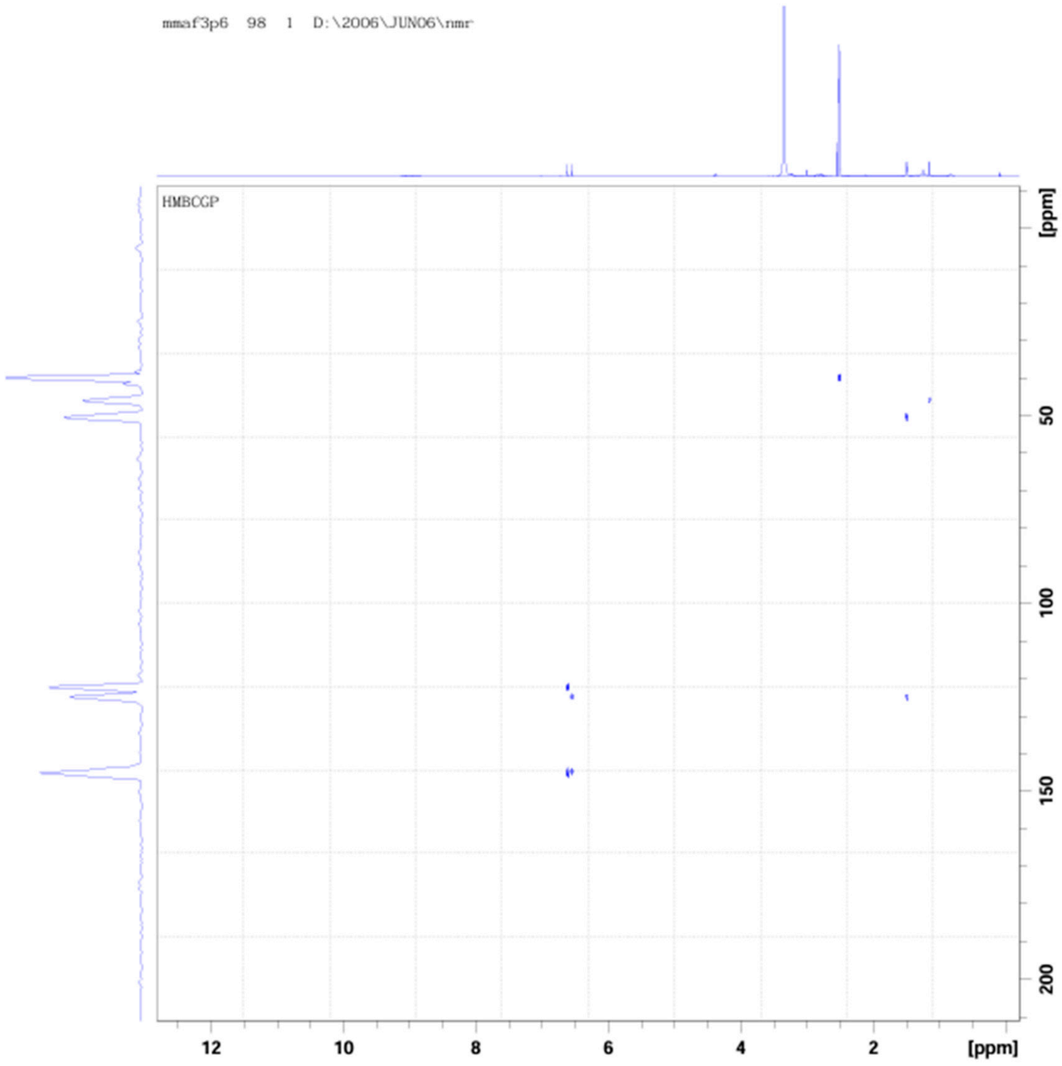

Figure S2. HMBC-NMR (500 MHz - DMSO) of G3 peak.

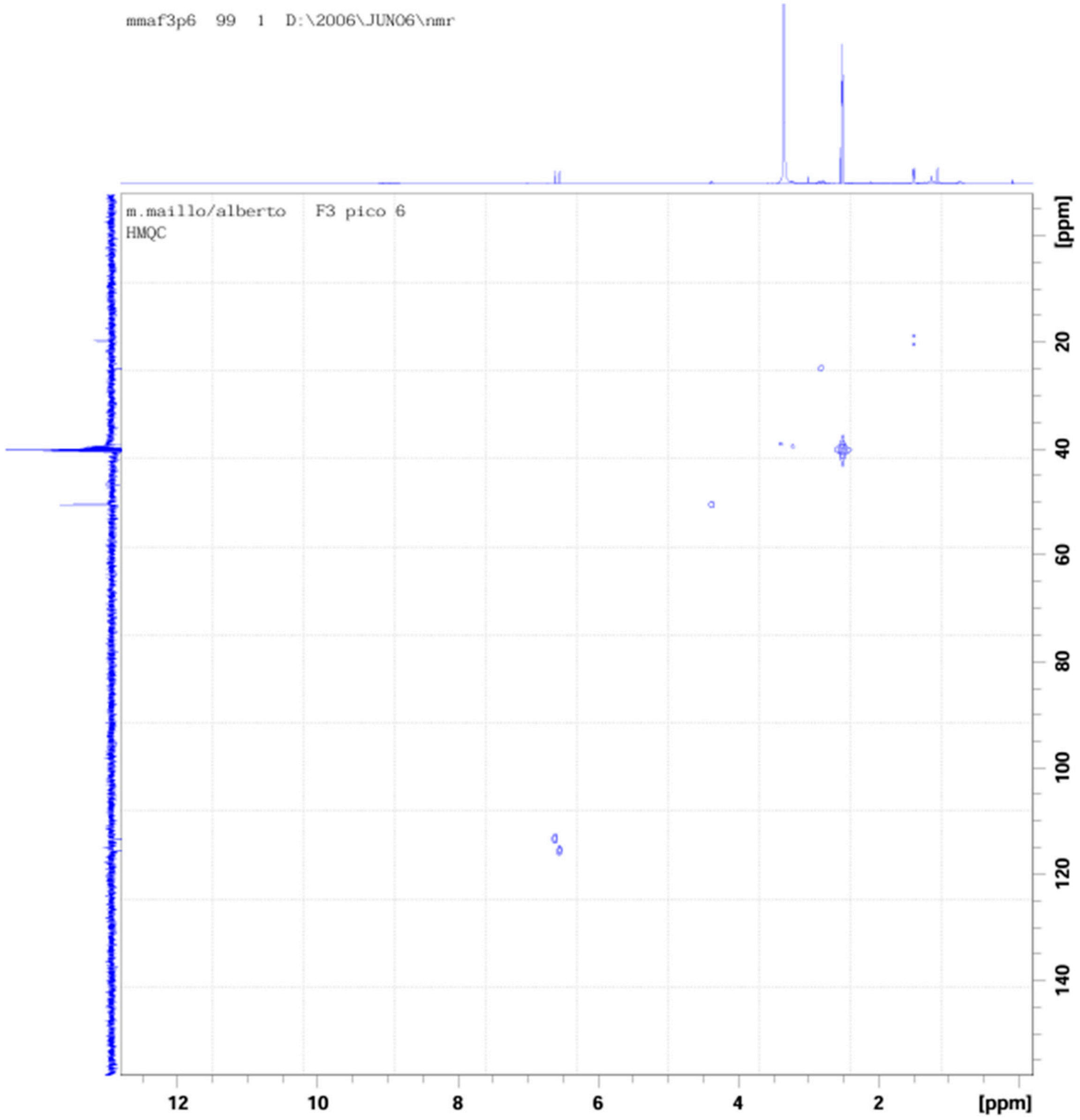

Figure S3. HMQC-NMR (500 MHz - DMSO) of G3 peak illustrating the proton-carbon correlation used for the total signal assignments.
